# Preparation and characterization of chemically cross-linked zwitterionic copolymer hydrogel for direct dye and toxic trace metal removal from aqueous medium

**DOI:** 10.1007/s11356-023-26966-7

**Published:** 2023-05-15

**Authors:** Asmaa A. Koryam, Shaimaa T. El-Wakeel, Emad K. Radwan, Azza M. Abdel Fattah, Elham S. Darwish

**Affiliations:** 1 Water Pollution Research Department, National Research Centre, 33 El Buhouth St, DokkiGiza, 12622 Egypt; 2 Department of Chemistry, Faculty of Science, University of Cairo, Giza, 12613 Egypt

**Keywords:** Polymeric hydrogel, Free radical polymerization, Adsorption, Wastewater treatment, Lead ions, Direct blue 71 dye

## Abstract

In this work, a zwitterionic copolymer hydrogel with adsorption affinity toward anionic dye and cationic trace metal was prepared by a free radical copolymerization of cationic ([3-(methacryloylamino)propyl] trimethylammonium chloride (MPTC)) and anionic (sodium 4-vinylbenzenesulfonate (SVBS)) monomers. Bis[2-(methacryloyloxy)ethyl] phosphate was used as a cross-linker and its effect on the adsorption properties of the prepared hydrogel was evaluated. The prepared materials were characterized by FTIR, XRD, SEM, EDX, and N_2_ adsorption at 77 K analysis. FTIR and EDX analysis demonstrated the successful preparation of poly(MPTC-co-VBS). XRD and SEM analysis showed that the poly (MPTC-co-VBS) is amorphous and has quasi-honeycomb morphology with large pores. Increasing the amount of the cross-linker enhanced the adsorption of direct blue 71 dye (DB71) and Pb(II) ions. The highest removal of DB71 and Pb(II) was achieved after 2 h using 1.5 g/L of poly(MPTC-co-VBS); however, the optimum solution pH was 3 for DB71 and 5 for Pb(II). The kinetics and isotherm studies illustrated that the surface of poly(MPTC-co-VBS) is heterogenous with small-sized homogenous pitches and the DB71 and Pb(II) adsorption onto poly(MPTC-co-VBS) is favorable. Finally, poly(MPTC-co-VBS) is more efficient in removing DB71 and Pb(II) from aqueous solutions than many other reported adsorbents.

## Introduction

Water is the source of life on Earth as it is the foundation of the food supply and the productive element to all livings (Chaplin 2001; Kılıç 2020). Over the last decades, increasing attention has been paid to water decontamination from trace metals and synthetic organic dyes due to their deteriorating effects on the quality of water resources (El-Naggar et al. 2022; Shaheen et al. 2022). Synthetic organic dyes are involved in many industries such as plastics, paper printing, textile dyeing, and cosmetics. Most of synthetic organic dyes are highly hazardous and toxic to human and aquatic environment due to their complex structures which make them stable and difficult to degrade. The presence of synthetic organic dyes in water resources even in trace amount leads to a decline in water quality and serious health problems (Crini 2003; Yang et al. 2021). Similar to the synthetic organic dyes, trace metals are toxic and hazardous materials as well. Lead is one of the most dangerous and abundant metals. It originates from human activities such as burning fossil fuels, mining, and manufacturing (Hasegawa et al. 2011) and enters the body through inhalation of dust and fumes or ingestion of lead-contaminated water (Xie et al. 2020). It causes serious diseases especially for children such as mental retardation that arises from its sturdiness and biological complexibility in human body (Tiwari et al. 2013).

Several methods have been developed to remove synthetic organic dyes and trace metals from aquatic environment. Adsorption has become a hot research topic and the most widely used technique for the removal of trace metals and synthetic organic dyes owing to its merits of simplicity, cost-effectiveness, ease of operation, possibility of reuse, and high efficiency (Abbas and Kassm 2021; van Kuringen et al. 2014).

Lately, the interest in the synthesis of zwitterionic hydrogels for water treatment has been growing. Zwitterionic hydrogels are three-dimensional networks of zwitterionic polymer or copolymers (Wei et al. 2018). They are highly polar and hydrophilic due to their unique structure that contains both anionic and cationic functional groups. In addition, zwitterionic hydrogels, by virtue of their unique chemical structure, can interact with both positive and negative contaminants via several mechanisms including electrostatic attractions, coordination bonding, and hydrogen bonding. The charge of the zwitterionic hydrogels can be controlled by the pH of the solution and salt concentration which empower selective removal of oppositely charged contaminants from a complex water matrices (Qu et al. 2022). Therefore, many zwitterionic hydrogels have been applied in water treatment as adsorbents for toxic trace metals and synthetic organic dyes. For example, Rehman et al. (2019) synthesized poly(3-acrylamidopropyl)-trimethylammoniumchloride-co-2-acrylamido-2-methylpropanesulphonic acid zwitterionic copolymer hydrogel and used it for the adsorption of crystal violet and Congo red from aqueous medium. In another study, Wei et al. (2018) synthesized poly(methylacryloyloxyethyl trimethylammonium chloride-co-acrylic acid zwitterionic copolymer hydrogel and tested its efficiency for the removal of both cationic dyes (methylene blue and methyl violet) and anionic dyes (amaranth and quinolone yellow) at different pH values.

In this work, we aimed at synthesizing a zwitterionic copolymer hydrogel (ZCPH) by the free radical co-polymerization of sodium 4-vinylbenzenesulfonate (SVBS) and [3-(methacryloylamino) propyl] trimethylammonium chloride (MPTC) for the removal of toxic trace metals and synthetic dyes from water. Ammonium persulfate (APS) was used as the initiator and bis[2-(methacryloyloxy) ethyl] phosphate (BMEP) as the cross-linker. Different amounts of the cross-linker were used to evaluate its effect on the adsorption properties of the resulting ZCPH toward trace metals and anionic synthetic organic dyes using lead (II) ions and direct blue 71 (DB71) as representatives for trace metals and anionic synthetic organic dyes, respectively. Among the different synthesized ZCPH, the adsorption properties of the most promising one was investigated in details. Specifically, the factors affecting the adsorption efficiency such as initial pH (pH_o_) of the adsorptive solution, amount of the synthesized ZCPH, and contact time were investigated. Finally, the adsorption kinetics and equilibrium data were analyzed by different models.

## Materials and methods

### Materials

Sodium 4-vinylbenzenesulfonate (≥ 90%), [3-(methacryloylamino) propyl] trimethylammonium chloride solution (50 wt. % in H_2_O), bis[2-(methacryloyloxy)ethyl] phosphate, ammonium persulfate (98%), direct blue 71, sodium chloride (NaCl), and lead(II) nitrate (Pb(NO_3_)_2_) were purchased from Sigma-Aldrich and used as received.

### Synthesis of poly (MPTC-co-VBS)

Three samples of poly (MPTC-co-VBS) with different amounts of the cross-linker (BMEP) were prepared by a free radical random copolymerization method to evaluate the effect of cross-linker on the adsorption properties of the resulting ZCPH. In brief, definite amounts of the monomers (MPTC and SVBS) and the cross-linker were first completely dissolved in deionized water (DIW) and purged with N_2_ for 15 min. Afterward, the initiator (APS) was added and the stirring and N_2_ purging were continued for additional 15 min. The mol percent of APS to the monomers was fixed at 1. The polymerization reaction was initiated by placing the mixture in an oven at 60 °C. After 24 h, the formed gel was washed with ethanol and DIW several times to remove the unreacted species and undesirable by-products then freeze dried using Labconco freeze dryer, USA. The exact amounts of the monomers, cross-linker, and initiator used in the preparation of the different samples and the code of the resulting samples are given in Table 1.

**Table 1:** Code of the prepared poly(MPTC-co-VBS) samples and their exact composition

Sample code	MPTC (mL)	SVBS (g)	BMEP (µL)	APS (mg)	DIW (mL)
ZCP	4.0	2.05	0	44.5	38.0
ZCPH-1	4.0	2.05	49.0	44.5	38.0
ZCPH-2	4.0	2.05	98.0	44.5	38.0

### Characterization of poly(MPTC-co-VBS)

The functional groups of the synthesized polymers were determined by Fourier transform infrared spectrometer Jasco FT/IR-47, while the crystal structure was characterized by an X-ray diffractometer (PANalytical X'Pert Pro) between 2θ of 5 and 60°. The morphology and elemental composition of the synthesized polymer were observed by JEOL 6400 F field emission scanning electron microscopy (FESEM) with energy dispersive X-ray analysis (EDX). The porous structure was determined by measuring N_2_ adsorption/desorption at 77 K using BELSORP-max surface analyzer. The pore size and pore size distribution were determined using the non-local density functional theory/grand canonical Monte Carlo (NLDFT/GCMC) method and the specific surface area and total pore volume were determined using the Brunauer, Emmett, and Teller (BET) method.

The pH of the point of zero charge (pH_pzc_) of the most promising ZCPH was determined following the salt addition method (Bakatula et al. 2018). In brief, 0.25 g of the hydrogel was added to a series of 50 mL of 0.01 M NaCl solutions preadjusted to different pH_o_ values and shaken at room temperature. After 24 h, the final pH was measured and the changes in the solution pH (ΔpH) values were calculated and plotted versus pH_o_. The pH_pzc_ was identified as the point of intersect of the curve with the pH_o_ axis.

### Adsorption method

**Fig. 1:**
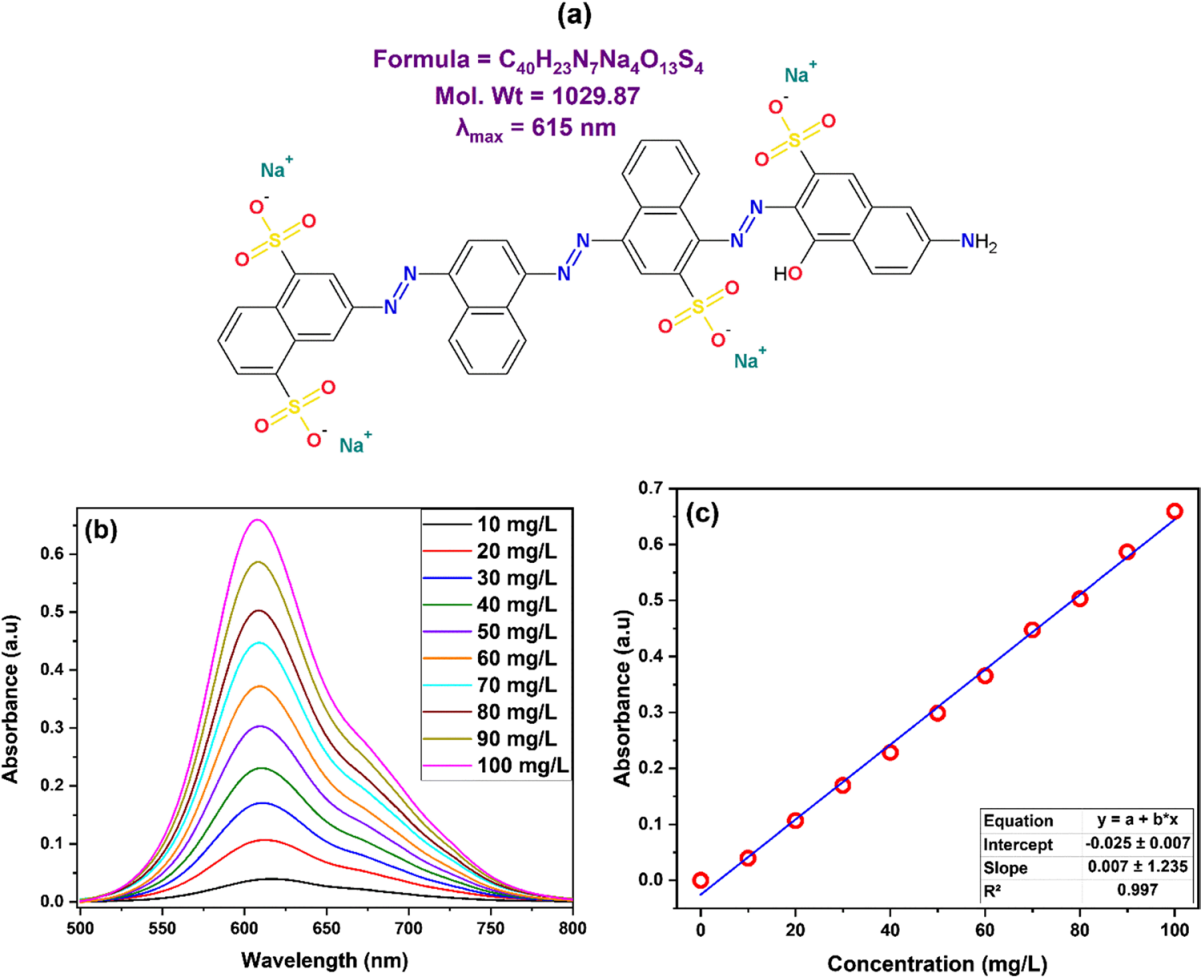
**a** Structure and selected properties, **b** visible spectra of different concentrations, and **c** calibration curve of DB71 dye

DB71 was used as a model for synthetic organic dyes. DB71 is an anionic direct azo dye that has three azo groups. Figure 1a gives the structure, formula, and molecular weight of the dye. The effect of pH_o_ of DB71 or Pb(II) solutions, mass of polymer, and initial concentration of DB71 or Pb(II) solutions on the adsorption efficiency of the most promising ZCPH (ZCPH-2) was tested in a single-component batch mode. All experiments were conducted in 250-mL conical flasks at room temperature using a speed controllable orbital shaker. Samples were withdrawn at different time intervals, then filtered and the remaining concentration of DB71 or Pb(II) was determined. Jasco V730 was used to determine the concentration of DB71 dye. First, the visible spectrum of a series of DB71 solutions with different concentrations was measured (Fig. 1b). The characteristic absorbance peak of DB71 was found at 615 nm. Then, a ten-point calibration curve (Fig. 1c) was constructed and used for the determination of DB71 concentration. Lead (II) ions concentration was determined using inductively coupled plasma optical emission spectrometry (ICP-OES, Agilent 5100).

The amount of DB71 or Pb(II) ions adsorbed onto a gram of the polymer (*q*_*t*_ (mg/g)) after contact time *t* (min) and the removal percentage (R%) were calculated using Eqs. 1 and 2, respectively.

1 $${\text{q}}_{\text{t}}\text{ = }\left({\text{C}}_{\text{o}} \text{- }{\text{C}}_{\text{t}}\right)\frac{\text{V}}{{\text{m}}}$$

2 $$\mathrm{R}\% = \left(\frac{C_{o}-C_{t}}{C_{o}}\right)100$$

where *C*_*o*_ (mg/L) is the initial concentration, *C*_*t*_ (mg/L) is the concentration after contact time t, V (L) is the volume of adsorptive solution, and m (g) is the mass of polymer.

The kinetic data was analyzed using the pseudo-first-order (PFO, Eq. 3) (Langergren and Svenska 1898), pseudo-second-order (PSO, Eq. 4) (Blanchard et al. 1984), and Elovich (Eq. 5) (Roginsky and Zeldovich 1934) models. The PFO model supposes that the rate of adsorption depends on the concentration of the adsorptive and controlled by the diffusion process.

3 $$\mathbf{PFO}\;\mathbf{model}\qquad\quad{{q}}_{{t}}{ = }{{q}}_{{e}}{ (}{1 - }{{e}}^{-{{k}}_{1}{{t}}}{)}$$

where *q*_*e*_ (mg/g) is the adsorption capacity at equilibrium, and *k*_*1*_ (min^−1^) is the PFO rate constant.

The PSO model provides accurate description of adsorption processes that is controlled by a chemical reaction between the adsorbent and the adsorptive, i.e., the surface chemisorption is the rate-limiting step.

4 $$\mathbf{P}\mathbf{S}\mathbf{O}\;\mathbf{m}\mathbf{o}\mathbf{d}\mathbf{e}\mathbf{l}\qquad\quad q_{t}=\frac{k_{2}q_{e}^{2}t}{1+{k}_{2}q_{e}t}$$

where *k*_*2*_ (g/mg min) is the PSO rate constant.

Elovich model describes the activated chemical adsorption and is suitable for adsorbents with heterogeneous adsorption sites.

5 $$\mathbf{Elovich}\;\mathbf{model}\qquad\quad q_{t} = \frac{1}{\beta} \text{ln}(1 + \alpha \beta t)$$

where *β* (g/mg) and *α* (mg/(g min)) are constants related to degree of surface coverage and rate of chemisorption, respectively.

The adsorption equilibrium data were analyzed using the Langmuir (Eq. 6) (Langmuir 1918) and Freundlich (Eq. 7) (Freundlich 1906) models. Langmuir model describes monolayer adsorption onto the surface of an adsorbent that has finite number of identical adsorption sites, it can be written as:

6 $$\mathbf{Langmuir}\;\mathbf{model}\qquad\quad q_{e} = \frac{q_{L}K_{L}C_{e}}{1 + K_{L}C_{e}}$$

where *K*_*L*_ (L/mg) is the Langmuir adsorption constant and q_L_ (mg/g) is the theoretical monolayer adsorption capacity.

Freundlich model is an empirical model that is used to describe adsorption onto an adsorbent that has heterogeneous adsorption sites, i.e., the adsorption sites has non-ideal distribution of adsorption heat and affinities, and it is not restricted to the formation of monolayer.

7 $$\mathbf{Freundlich} \; \mathbf{model} \qquad\quad q_{e} \, = K_{F} C_{e}^{1/}n$$

where *K*_*F*_ is a constant (mg^(1–1/*n*)^L^(1/*n*)^/g) related to adsorption capacity and adsorption intensity.

The separation factor (R_L_) (Eq. 8) is an essential characteristic of Langmuir model which indicates whether the adsorption is favorable (0˂R_L_˂1), unfavorable (R_L_˃1), linear (R_L_ = 1), or irreversible (R_L_ = 0).

8 $$\mathbf{S}\mathbf{e}\mathbf{p}\mathbf{a}\mathbf{r}\mathbf{a}\mathbf{t}\mathbf{i}\mathbf{o}\mathbf{n}\;\mathbf{f}\mathbf{a}\mathbf{c}\mathbf{t}\mathbf{o}\mathbf{r}\;\;\;\;\;\;\;\;\;\;\;\;\;\;\;\;{{R}}_{{L}}{ } = \frac{1}{{1+ }{K}_{L}{{ C}}_{{o}}}$$

The nonlinear fitting of the kinetics and isotherm models was conducted using OriginPro 2016 Ver. 9.3.226 software. The residual sum of squares was minimized using orthogonal distance regression algorithm regression method. The coefficient of determination (R^2^), chi-square (χ^2^), and the sum of squared estimate of errors (SSE) were calculated by the Origin software and used as a measure to compare models fitting, evaluate the precision of the predicted data, and define the model that best predicts the practical data (Abbas and Kassm 2021).

## Results and discussion

**Scheme 1:**
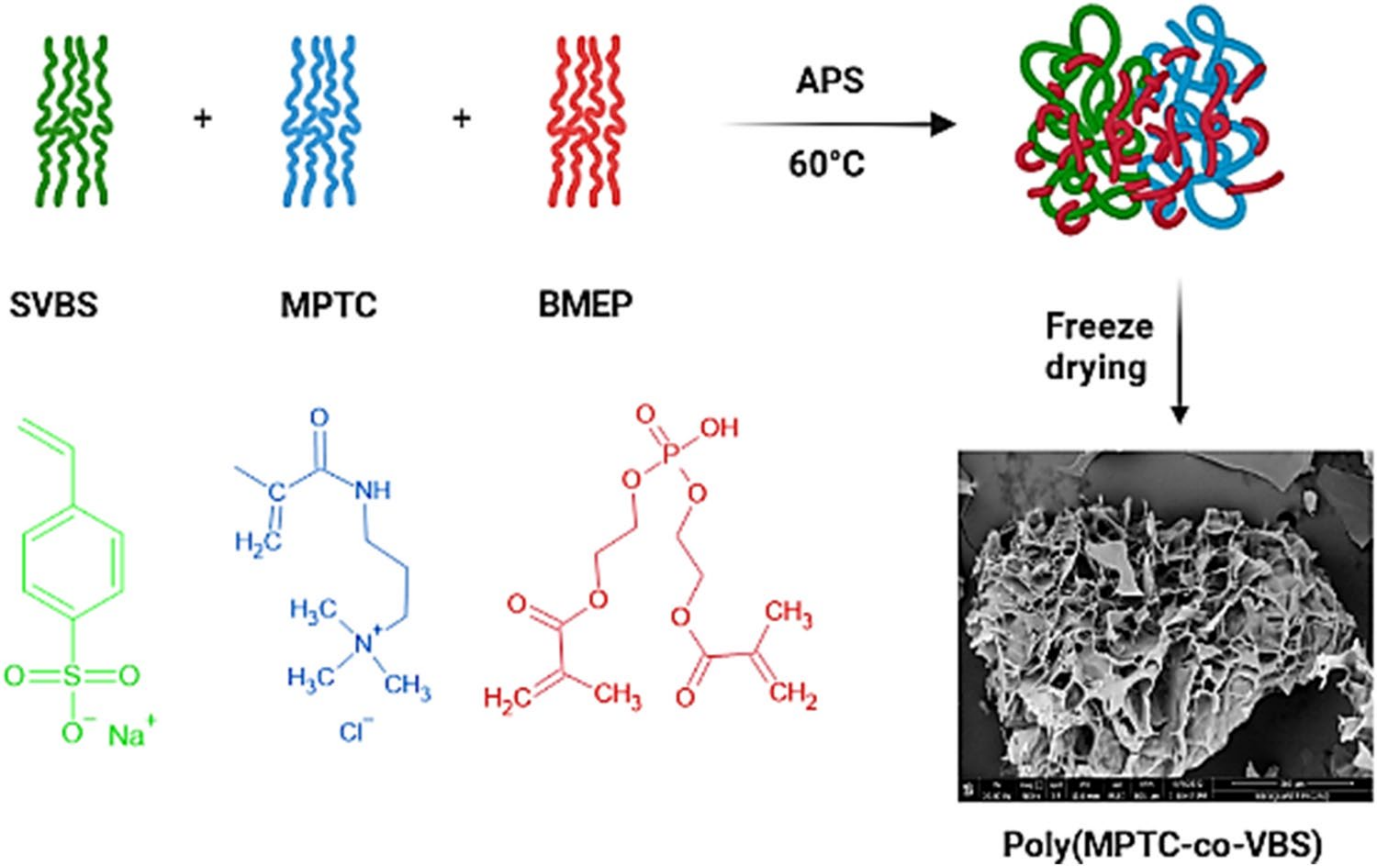
Synthetic procedure of poly(MPTC-co-VBS)

Lately, synthesis of new materials capable of removing both anionic and cationic pollutants from water is attracting excessive attention. In this work, we targeted the preparation of a new copolymer hydrogel that bears permanent anionic and cationic groups. Therefore, SVBS was selected as the anionic monomer because it contains a sulphonate group which keep its negative charge down to pH 1 (El Malah et al. 2021) and MPTC was selected as the cationic monomer because it contains a quaternary ammonium group which is positively charged at any pH. The zwitterionic poly(MPTC-co-VBS) hydrogel was prepared following a free radical random copolymerization reaction between SVBS and MPTC using BMEP as cross-linker and APS as initiator. Scheme 1 represents the preparation procedure.

BMEP is a phosphate ester that can act as a bifunctional cross-linker owing to its structure which consists of a central phosphate group and two terminal polymerizable methacrylate groups. BMEP was selected as the cross-linker owing to its numerous advantages including abundance of functional groups, biocompatibility, biodegradability, enzymatic degradability, and non-cytotoxicity. Additionally, the phosphate group of BMEP enhances the adsorption capacity of the hydrogel and makes it flexible and thermally stable (Anil et al. 2020; Nakhjiri et al. 2018). After the well dissolution and mixing of SVBS, MPTC, BMEP, and APS, the solution was heated at 60 °C to activate APS. Upon heating, APS decomposes into sulfate ion radicals which attack the double bonds of SVBS, MPTC, and BMEP to create new initiating polymer chains. The generated reactive polymer chains continue to propagate and crosslink leading to increasing the viscosity of the solution and eventually termination of the polymerization reaction and formation of gel. The Na^+^ of SVBS and Cl^−^ of MPTC form NaCl as an unwanted side product; therefore, washing the resulting gel with ethanol and DIW was necessary to get rid of the unreacted species and the formed NaCl.

### Characterization of poly(MPTC-co-VBS)

**Fig. 2:**
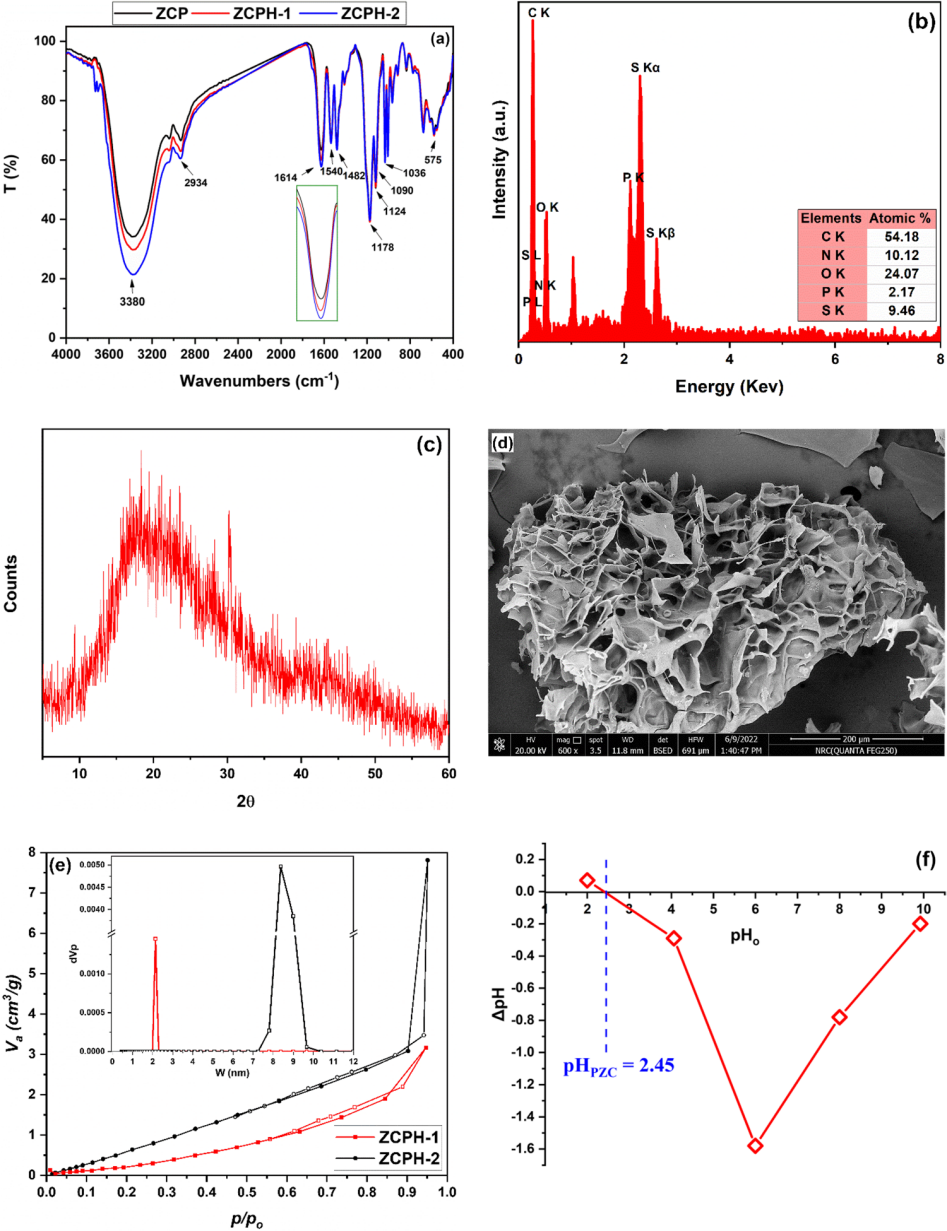
**a** FTIR spectra of the synthesized polymers, **b** N_2_ adsorption desorption curves of ZCPH-1 and ZCPH-2, and **c** EDX spectrum, **d** XRD pattern, **e** SEM image, and **f** pH_PZC_ of ZCPH-2

The FTIR of the synthesized polymers was conducted to investigate their chemical composition. Figure 2a illustrates that the FTIR spectra of the three synthesized polymers are very similar. The broad peak at around 3380 cm^−1^ can be assigned to –OH stretching of adsorbed water (He et al. 2017), while the C-H stretching peak appears near 2934 cm^−1^ (Tran et al. 2019). The peaks at 1482 cm^−1^ and 1540 cm^−1^ are assigned to in-plane bending of methyl groups of the quaternary ammonium group (-N^+^(CH_3_)) and the stretching of CON-H group, respectively, which originate from the MPTC monomer (Pourjavadi et al. 2013; Tran et al. 2019; Zhu et al. 2016). The peaks at 1120 and 1010 cm^−1^ are corresponding to the in-plane skeleton and bending vibration, respectively, of the phenyl group (Tran et al. 2019). The peaks at 1036 and 1178 cm^−1^ are corresponding to the antisymmetric and symmetric stretching of S = O in SO_3_^− ^group originating from VBS monomer (He et al. 2017; Tran et al. 2019). The band at 1614 cm^−1^ corresponded to the stretching vibration of C = O (He et al. 2017) of the cross-linker (BMEP). The inset of Fig. 2a shows that the peak of C = O becomes more intense as the amount of the cross-linker increased; the polymer ZCPH-2 has the most intense peak. The characteristic peaks of phosphate groups of the cross-linker (BMEP) appeared between 1090–1030 and 600–560 cm^−1^ (Lai et al. 2005). It can be noticed that the distinguishing absorption peaks of C = C at 1656 and 1629 cm^−1^ are absent indicating the participation of the vinyl groups of MPTC and VBS in the polymerization reaction to form poly(MPTC-co-VBS). Overall, the FTIR results obviously demonstrate the successful preparation of BMEP cross-linked poly(MPTC-co-VBS).

To further confirm the successful synthesis of BMEP cross-linked poly(MPTC-co-VBS), the elemental composition of ZCPH-2 was analyzed using EDX; the spectrum is shown in Fig. 2b. In addition to carbon (C) and oxygen (O), the EDX spectrum illustrated the presence of sulfur (S), nitrogen (N), and phosphorus (P) elements which indicates the incorporation of SVBS, MPTC, and BMEP, respectively, in the ZCPH-2 matrix.

The crystallinity and purity of the ZCPH-2 were investigated using XRD analysis. The XRD pattern displayed in Fig. 2c shows a broad hump around 2θ = 20° which cannot be ascribed to any crystalline model. Therefore, the synthesized ZCPH-2 is an amorphous polymer. The obtained XRD diffraction in this study is characteristic for amorphous polymers (Kundu and Bhaumik 2015; Subramani 2016). Noteworthy that the reaction of SVBS and MPTC results in the formation of NaCl as an undesirable by-product. Absence of diffraction peaks in the XRD pattern indicates the purity of the ZCPH-2 and the effectiveness of the washing step applied after the synthesis of the polymer in the removal of the formed NaCl.

The microstructure morphology of hydrogel is an important feature which defines its ability to absorb and retain water. Hence, the morphology of ZCPH-2 was captured by SEM and displayed in Fig. 2d. It is apparent that ZCPH-2 has a 3D heterogeneous quasi-honeycomb highly porous structure with large pores. This morphology is expected to facilitate the diffusion and penetration of pollutants from water into the pores of ZCPH-2. It is known that freeze-drying is one of the techniques that are used to prepare 3D porous materials (Fereshteh 2018; Grenier et al. 2019). During the freeze-drying process, the sublimation of the ice crystals leaves behind porous structure (Grenier et al. 2019).

To get more information about the porous structure of ZCPH-1 and ZCPH-2, the N_2_ adsorption–desorption isotherm and the NLDFT/GCMC pore size distribution curves were measured and are given in Fig. 2e. The N_2_ adsorption isotherms of both ZCPH-1 and ZCPH-2 have not knee point and are convex to the relative pressure axis; therefore, it can be assigned to type III of the International Union of Pure and Applied Chemistry (IUPAC) classification. Type III isotherm suggests that the interactions between N_2_ and ZCPH-1 or ZCPH-2 are relatively weaker than the interactions between the adsorbed N_2_ molecules, and that the adsorbed N_2_ molecules are gathered around the most favorable adsorption sites of ZCPH-1 and ZCPH-2. The hysteresis loops of both ZCPH-1 and ZCPH-2 belong to type H3 of the IUPAC classification. Type H3 loop is common for non-rigid agglomerations of plate-like materials (Sing 1985; Thommes et al. 2015). The NLDFT pore size distribution curves (inset of Fig. 2e) expose the mesoporous nature of both ZCPH-1 and ZCPH-2. Notably, ZCPH-2 has considerably wider pore sizes and higher pore volume than those of ZCPH-1 according to the NDLFT results. Similarly, BET results showed that ZCPH-2 has significantly higher surface area (5.05 m^2^/g) and total pore volume (12 cm^3^/g) than those of ZCPH-1 (2.54 m^2^/g and 5 cm^3^/g, respectively).

The point of zero charge is an important character which affects the adsorption properties of a material. It is defined as the pH value at which the positive charges (cations) density at the surface equals that of negative charges (anions) (Rey et al. 2017). Figure 2f shows the plot of ΔpH vs pH_o_ for ZCPH-2. It can be observed that the pH_PZC_ is 2.45. Therefore, at pH_o_ > 2.45, the net charge on the surface of ZCPH-2 is negative and it will behave as an anionic polyelectrolyte whereas at pH_o_ < 2.45, the net charge on the surface is positive and it will behave as a cationic polyelectrolyte.

### Adsorption properties of the synthesized poly(MPTC-co-VBS)

**Fig. 3:**
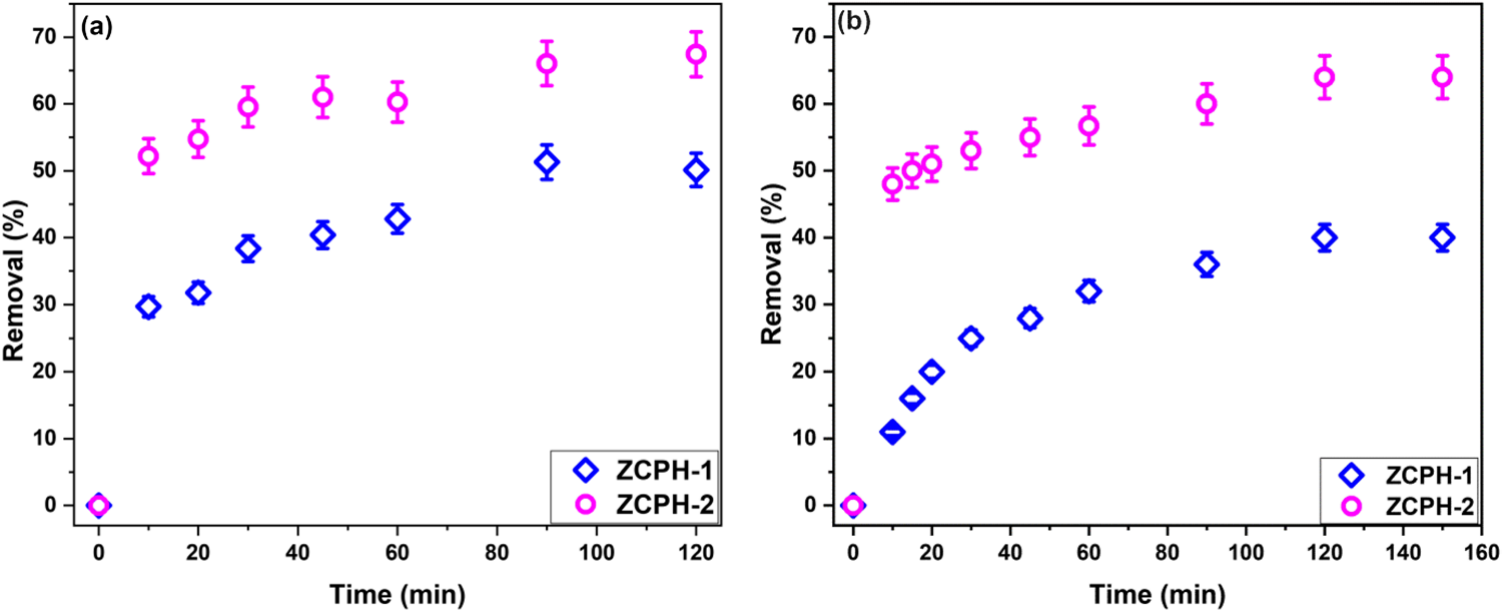
Comparison between ZCPH-1 and ZCPH-2 for the removal of **a** DB71 and **b** Pb(II) ions

The adsorption efficiency of the three synthesized polymers toward DB71 and Pb(II) was assessed and compared. It was found that the polymer prepared in absence of the cross-linker (ZCP) was water soluble. Therefore, it was excluded from further study. It is known that crosslinking water soluble polymers converts them into insoluble compounds (Rivas et al. 2018). Figure 3 represents the change of the percentage of DB71 and Pb(II) removed with time using 1 g/L of the two cross-linked polymers (ZCPH-1 and ZCPH-2). The initial concentration of both DB71 and Pb(II) was 10 mg/L, and the pH_o_ of the solutions was set at 5. Figure 3a shows that the R% of DB71 increased from 30 to 50% when the contact time increased from 10 to 90 min. When the amount of cross-linker increased, sample ZCPH-2, the R% of DB71 increased from 52 to 66% with progressing the contact time from 10 to 90 min. A similar behavior was observed for Pb(II) adsorption. Figure 3b shows that the R% of Pb(II) was 11% after 10 min and increased to 40% at 120 min. Increasing the amount of cross-linker, sample ZCPH-2, caused an increase in the R% of Pb(II) from 48% at 10 min and 64% at 90 min. Thus, for both DB71 and Pb(II), a considerable increase in the removal percentages was achieved when the cross-linker amount increased. This observation can be attributed to increasing the surface area, pore volume, and functional groups content with increasing the cross-linker amount as discussed above. Anil et al. (2020) reported a similar trend of improving the interaction between methylene blue dye and poly(vinylphosphonic acid)/bis[2-(methacryloyloxy)ethyl] phosphate hydrogel when the amount of the cross-linker increased and explained the results by increasing the functionalities with the cross-linker amount. In summary, comparing the adsorption efficiency of ZCPH-1 and ZCPH-2 illustrated that the ZCPH-2 has higher affinity toward DB71 and Pb(II) ions than ZCPH-1. Consequently, further experiments were performed using ZCPH-2.

**Fig. 4:**
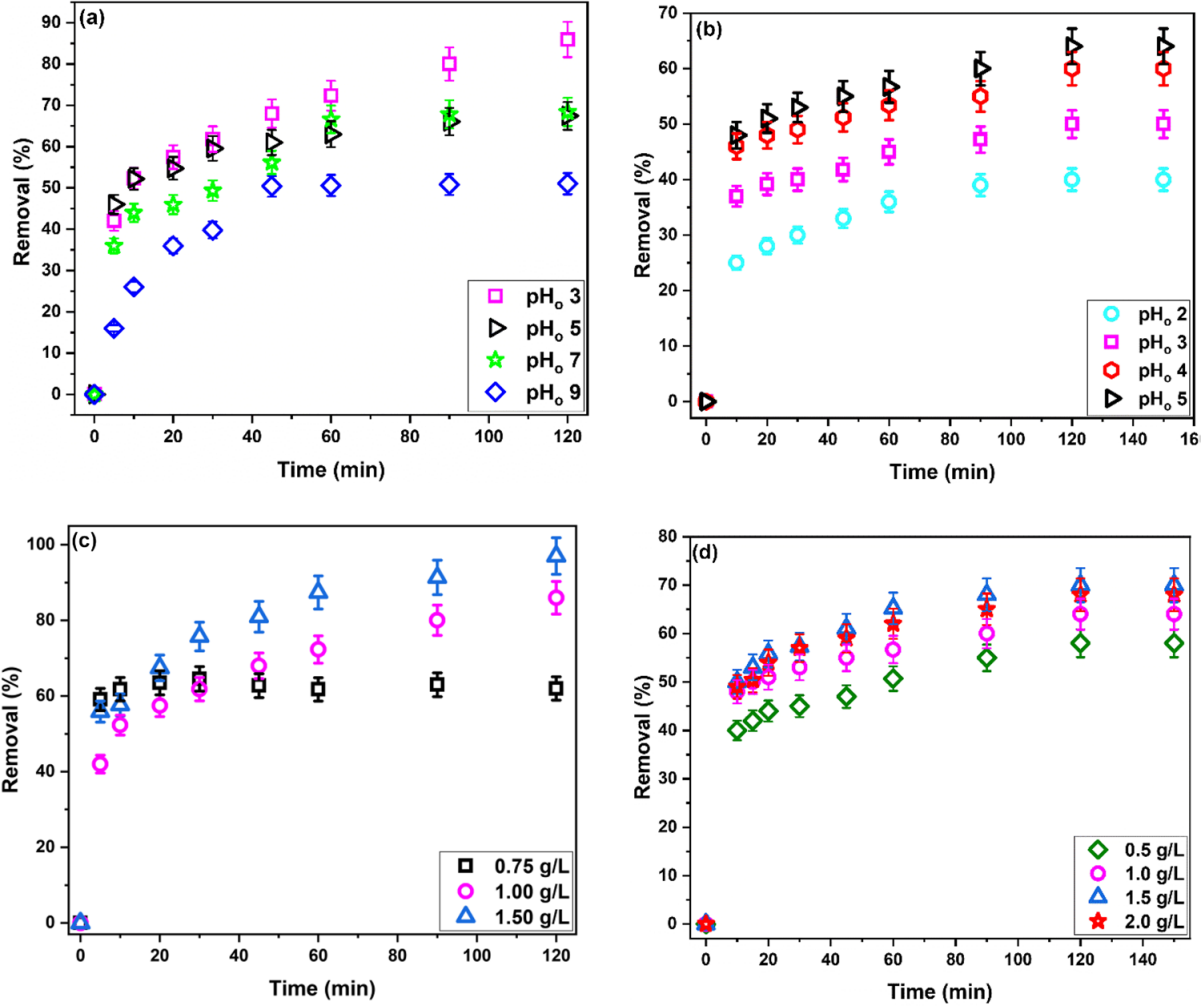
Effect of pH_o_ (**a** and **b**) and adsorbent dosage (**c** and **d**) on the removal of DB71 and Pb(II) ions, respectively, by ZCPH-2

Effects of pH_o_ on the adsorption of DB71 dye and Pb(II) onto ZCPH-2 were evaluated by contacting 10 mg/L of DB71 or Pb(II) solution pre-set at specific pH_o_ values with 1 g/L of ZCPH-2. The pre-designed pH_o_ value was attained by adding limited amounts of dilute solutions of HCl and NaOH. The results are illustrated in Fig. 4. For DB71 (Fig. 4a), the highest R% was achieved at pH_o_ 3 (85%) and decreased as the solution acidity decreased (67% at pH_o_ 5 and 68% at pH_o_ 7) reaching its minimum value (50%) at pH_o_ 9. The sulphonate groups of the DB71 render the dye anionic character owing to their weak base nature which make them stable anion even at pH 1 (El Malah et al. 2021). Meanwhile, the pH_PZC_ indicates that ZCPH-2 has a net negative charge at pH_o_ ˃2.45. At pH_o_ 3, the relatively low magnitude of negative charge allows the electrostatic attraction between DB71 and ZCPH-2 causing a higher R%. As the pH_o_ increased, the magnitude of the negative charge increases and the electrostatic repulsion between DB71 and ZCPH-2 progressively increases; meanwhile, the competition between the hydroxyl ions and DB71 for the adsorption sites of ZCPH-2 increases causing a decrease in R%. The observed removal at pH_o_ ˃ pH_PZC_ suggests that electrostatic attraction is not the sole mechanism underlying the adsorption process. The observed removal at pH_o_ ˃ pH_PZC_ can be ascribed partially to the electrostatic attractions between the quaternary ammonium group of the MPTC and the anionic dye. Another probable reason is the presence of another type of interactions other than the electrostatic interactions. Based on the structure of both DB71 and ZCPH-2, pore filling, hydrogen bonding, *n–π* interactions, and *π–π* interactions are possible. The SEM image (Fig. 2d) revealed the porous nature of ZCPH-2; therefore, pore filling might be a possible route for the removal of DB71. Also, ZCPH-2 contains amide, phenyl and sulfonate groups, while DB71 contains amino and hydroxyl groups in addition to the sulfonate and azo groups. The amine group of the DB71 can bind to the sulfonate group of ZCPH-2 via dipole–dipole hydrogen bonding. Similarly, dipole–dipole hydrogen bonding can be formed between the sulfonate group of DB71 and the amide group of ZCPH-2. Yoshida hydrogen bonding can take place between the amine group of the DB71 and phenyl groups of ZCPH-2 and also between naphthalene rings of DB71 and amide group of ZCPH-2. *n–π* electron donor–acceptor interactions can occur between the amine group of DB71 and the phenyl group of ZCPH-2 and also between the amide group of ZCPH-2 and naphthalene rings of DB71. Finally, the phenyl groups of ZCPH-2 and naphthalene rings of DB71 can generate *π–π* stacking interactions. Overall, the adsorption of DB71 onto ZCPH-2 occurs through one or more of these interactions and probable synergism among them.

Figure 4b shows that the adsorption of Pb(II) ion onto ZCPH-2 was low (40%) at pH_o_ 2 and increased as the acidity of Pb(II) solution decreased reaching its highest value (64%) at pH_o_ 5. The ZCPH-2 contains amide and sulfonate groups. The former group can be affected by the solution pH while the latter is stable anion even at pH 1 (El Malah et al. 2021). Thus, the sulfonate group drives the adsorption of Pb(II) at all the studied pH_o_ values. However, according to the value of pH_PZC_, the surface of ZCPH-2 carries a net positive charge at pH_o_ 2. Electrostatic repulsion between the Pb(II) cations and the positively charged ZCPH-2 hinders the approach of Pb(II) to the adsorption sites causing the low R% at pH_o_ 2. Above the pH_PZC_ (pH_o_ 2.45), the net charge of the surface of ZCPH-2 becomes negative; therefore, electrostatic interactions between the Pb(II) cations and the negatively charged ZCPH-2 take place and R% increases. Meanwhile, as the acidity of the solution decreases, the amide group of ZCPH-2 becomes available for Pb(II) adsorption consequently the R% increases. Notable that the R% at pH_o_ 5 (64%) was insignificantly higher than that at pH_o_ 4 (60%); however, pH_o_ 5 is privileged because it is closer to the neutral pH. Further increase in the pH_o_ is not recommended as it is known that Pb(II) ions start to precipitate as hydroxide at pH > 6 (Escudero-García et al. 2013). To sum up, the results of pH_o_ effects on Pb(II) adsorption point out that the interaction between Pb(II) ions and ZCPH-2 is mainly governed by electrostatic interaction.

Different amounts of ZCPH-2 were tested to evaluate the effects of the amount of ZCPH-2 on the R% of DB71 and Pb(II). These experiments were conducting using a solution of 10 mg/L DB71 or Pb(II) pre-set at pH_o_ 3 and 5, respectively. Figure 4c and d display the results. For both DB71 and Pb(II), increasing the amount of ZCPH-2 causes an increase in the R%. Particularly, the R% of DB71 increased from 62 to 86% then to 97% when the amount of ZCPH-2 increased from 0.75 to 1.00 g/L then 1.50 g/L. Similarly, the R% of Pb(II) increased from 58 to 64% then to 70% when the amount of ZCPH-2 increased from 0.50 to 1.00 g/L then 1.50 g/L. Increasing the amount of ZCPH-2 more than 1.50 g/L has minor effect on the R% of Pb(II), the R% was 68% using 2.00 g/L. Normally, increasing the amount of the adsorbent ensures increasing the adsorption sites, thus increasing the R%. Nevertheless, in some cases, increasing the adsorbent amount to certain levels leads to its agglomeration. The agglomeration of adsorbent results in concealment of some adsorption sites and increasing the diffusion path length consequently decreasing the R% (Alene et al. 2021; El-Naggar et al. 2022; Giri et al. 2022; Igwegbe et al. 2021; Radwan et al. 2022).

**Fig. 5:**
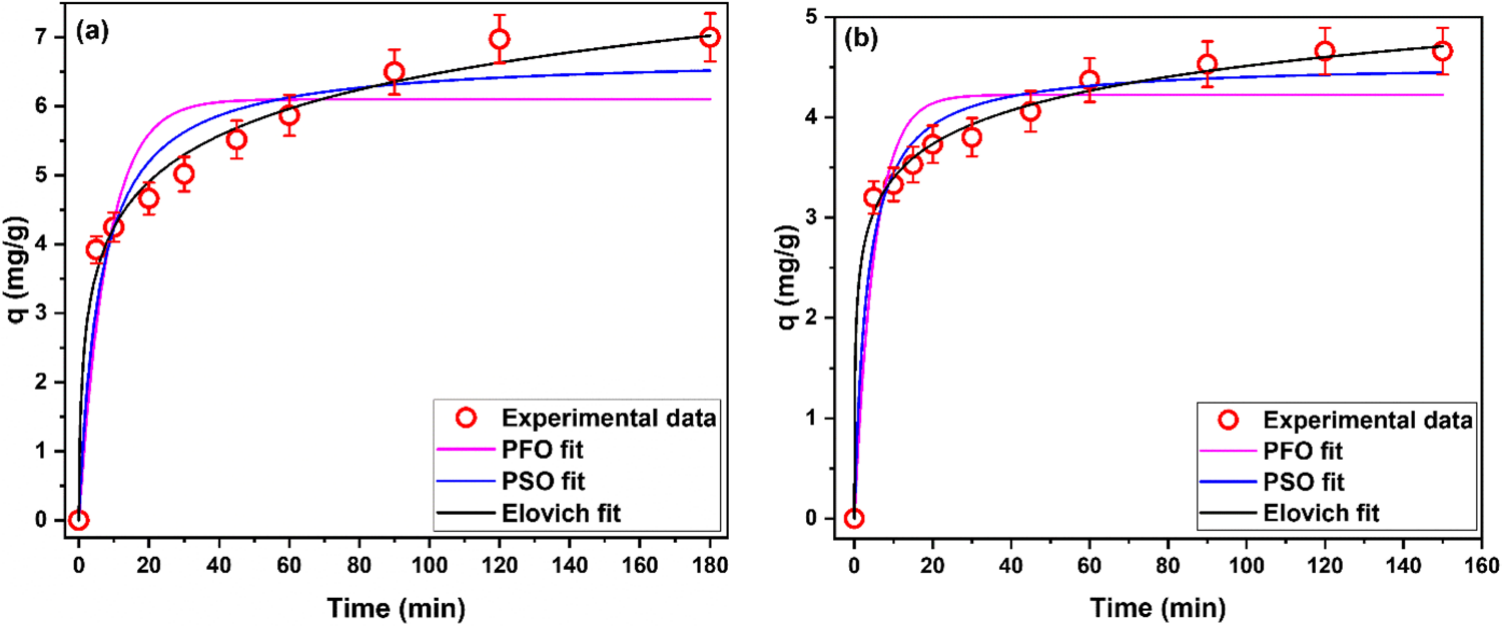
Experimental kinetics and fitted models for the adsorption of (**a**) DB71 (pH_o_ 3, dosage 1.5 g/L) and (**b**) Pb(II) ions (pH_o_ 5, dosage 1.5 g/L) onto ZCPH-2

Kinetic analysis is used to identify the rate and controlling mechanism of the adsorption process. In this study, PFO, PSO, and Elovich models were used to analyze the kinetics of DB71 and Pb(II) adsorption onto ZCPH-2. Figure 5 gives the time-profile of DB71 and Pb(II) adsorption onto ZCPH-2 as well as the fitting curves of the used kinetic models. The calculated kinetic parameters and error values are summarized in Table 2. Figure 5 shows that the amount adsorbed of DB71 and Pb(II) increased gradually as the contact time passed and attained the equilibrium state in 120 min for DB71 and 90 min for Pb(II).

**Table 2:** Calculated kinetic parameters and error functions for DR71 and Pb(II) ions

DR71					
PFO		PSO		Elovich	
*R*^2^	0.99989	*R*^2^	0.99995	*R*^2^	0.99999
*χ*^2^	0.61	*χ*^2^	0.26	*χ*^2^	0.05
SSE	4.88	SSE	2.11	SSE	0.42
q_e1_	6.10 ± 0.33	q_e2_	6.73 ± 0.31	*α*	7.68 ± 2.81
k_1_	0.12 ± 0.03	k_2_	0.03 ± 0.01	*β*	1.03 ± 0.08
Pb(II)					
PFO		PSO		Elovich	
*R*^2^	0.99996	*R*^2^	0.99999	*R*^2^	0.99999
*χ*^2^	0.16	*χ*^2^	0.06	*χ*^2^	0.008
SSE	1.46	SSE	0.52	SSE	0.07
q_e,1_	4.22 ± 0.15	q_e,2_	4.54 ± 0.13	*α*	53.56 ± 21.36
k_1_	0.19 ± 0.04	k_2_	0.07 ± 0.02	*β*	2.06 ± 0.11

Comparing the values of the correlation coefficient indicates that, for both DB71 and Pb(II), all studied kinetic models can describe the adsorption process. However, the values of error functions tell that PSO model fits the data better than the PFO model and Elovich model can describe the experimental data more accurately than the other two models. This result indicates that the adsorption process does not follow a simple mechanism. However, both PSO and Elovich models assume that chemical adsorption is the controlling mechanism of the adsorption process (Blanchard et al. 1984; Roginsky and Zeldovich 1934). In addition, Elovich model assumes that the adsorbent has heterogenous adsorption sites (Roginsky and Zeldovich 1934). Therefore, the kinetic analysis study indicates that ZCPH-2 has energetically heterogeneous adsorption sites and that the adsorption of DB71 and Pb(II) onto ZCPH-2 follows more than one mechanism; however, chemisorption is the predominate one. This result is logical owing to the existence of a variety of adsorption sites (pores, and quaternary ammonium, sulfonate, phenyl, and amide functional groups) onto ZCPH-2.

**Fig. 6:**
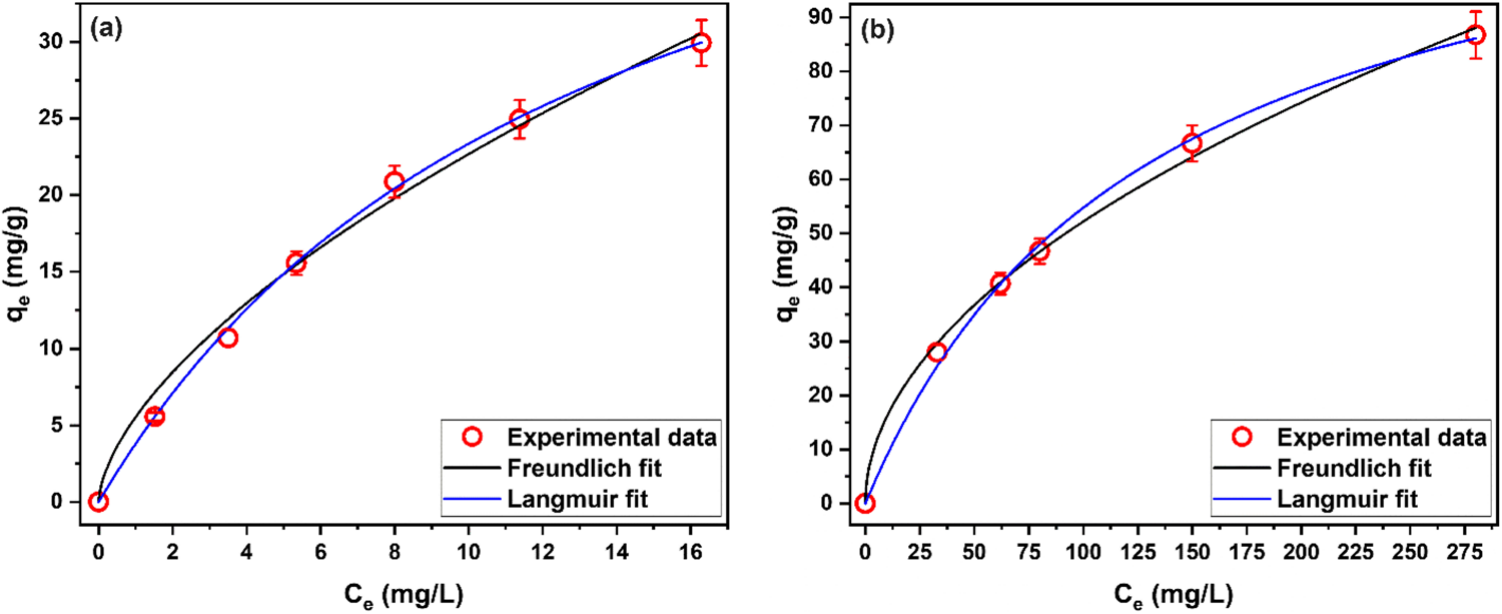
Experimental adsorption isotherm and fitted models for (**a**) DB71 (pH_o_ = 3, dosage = 1.5 g/L) and (**b**) Pb(II) ions (pH_o_ = 5, dosage = 1.5 g/L) uptake by ZCPH-2

Isotherm studies are used to recognize the characteristics of the adsorbent surface, the affinity of the adsorbent towards an adsorptive, and the nature of adsorbent–adsorbate interactions, and to evaluate the performance of the adsorbent and compare it to others. In this study, the adsorption equilibrium data was analyzed using the most common Langmuir and Freundlich isotherm models. Figure 6 gives the experimental adsorption isotherm and the fitted curves of Langmuir and Freundlich isotherm models.

The obtained values of the parameters and error functions of Langmuir and Freundlich models for both DB71 or Pb(II) are presented in Table 3. The values of correlation coefficient are higher than 0.99 for both models indicating that both models can describe the adsorption equilibrium data of DB71 or Pb(II). This result supports the finding of kinetic study that the adsorption does not follow a simple mechanism. Several researches have previously reported the fit of their equilibrium adsorption data to more than one isotherm model (El Malah et al. 2021; Lin et al. 2020; Ong et al. 2020; Song et al. 2020).

**Table 3:** Calculated isotherm parameters and error functions for DR71 and Pb(II) ions

Freundlich		Langmuir	
DR71			
*R*^2^	0.9904	*R*^2^	0.9999
*χ*^2^	0.22	*χ*^2^	0.02
RSS	1.11	RSS	0.11
K_F_	5.54 ± 0.55	K_L_	0.07 ± 0.00
*N*	1.63 ± 0.11	q_m_	54.44 ± 1.58
		R_L_	0.19–0.59
Pb(II)			
*R*^2^	0.9984	*R*^2^	0.9998
*χ*^2^	2.56	*χ*^2^	1.81
RSS	10.24	RSS	7.24
K_F_	5.02 ± 0.51	K_L_	0.01 ± 5.76 × 10^−4^
*N*	1.97 ± 0.08	q_m_	126.43 ± 4.47
		R_L_	0.25–0.67

The good fit of the equilibrium data to both Langmuir and Freundlich suggests that ZCPH-2 has a complex heterogenous surface with small-sized homogenous patches. These patches do not interact with each other and each patch has adsorption sites with equal energy. This suggestion looks realistic as the repeated units of MPTC have adsorption sites with homogenous adsorption energy. Similarly, the repeated units of VBS also have adsorption sites with homogenous adsorption energy. Therefore, the repeated units each of MPTC and VBS can be considered the homogenous patches. Meanwhile, a patch of MPTC has adsorption sites with adsorption energy different than those of a VBS patch which makes the surface of ZCPH-2 has a heterogeneous nature.

Analysis of the models parameters displayed in Table 3 shows that, for both DR71 and Pb(II), the values of Freundlich’s exponent (*n*_*F*_) were > 1, and the Langmuir separation factor (R_L_) was in the range of 0–1 which indicate that the adsorption of DR71 and Pb(II) onto ZCPH-2 is favorable. In order to evaluate the adsorption performance of ZCPH-2 relative to other reported adsorbents, the values of Langmuir theoretical monolayer saturation capacity of several adsorbents reported in the literature are collected in Table 4.

**Table 4:** Langmuir adsorption capacity reported for DB71 and Pb(II) adsorption by other adsorbents

DB71		Pb(II)	
Adsorbent	*q*_L_ (mg/g)	Adsorbent	*q*_L_ (mg/g)
Magnetic nanocomposite of chitosan/SiO_2_/CNTs (Abbasi 2017)	61	This work	126
This work	54	Carbon nanotubes (Kabbashi et al. 2009)	102
Mixed silica–alumina oxide (Wawrzkiewicz et al. 2015)	49	Xanthate-modified magnetic chitosan (Zhu et al. 2012)	77
Montmorillonite (Yavuz and Aydin 2006)	48	Lobeira fruit (Araújo et al. 2018)	51
Chitosan-MWCNTs (Abbasi and Habibi 2016)	29	Mustard husk (Meena et al. 2008)	30
Hazelnut shell based activated carbon (Yavuz and Aydin 2006)	26	Fe nanoparticles loaded ash (Ghasemi et al. 2014)	30
Raw kaolinite (Yavuz and Aydin 2006)	22	Celtek clay (Sarı et al. 2007)	18
Spent mushroom waste (Alhujaily et al. 2020)	20	Nanocomposite of carbon nanotubes/silica nanoparticles (Saleh 2016)	13
CPB modified zeolite (Mirzaei et al. 2018)	14	Kaolinite (Et and Shahmohammadi-Kalalagh 2011)	8
Nickel ferrite nanoparticles supported on clinoptilolite zeolite (Reza 2018)	6	Banana peels (Anwar et al. 2010)	2.18

It can be noticed from Table 4 that ZCPH-2 has higher adsorbent capacity towards Pb(II) compared to other adsorbents such as carbon nanotubes (Kabbashi et al. 2009), xanthate-modified magnetic chitosan (Zhu et al. 2012), and Lobeira fruit (Araújo et al. 2018). Likewise, the adsorption capacity of ZCPH-2 toward DB71 is higher than several other adsorbents such as chitosan-MWCNTs (Abbasi and Habibi 2016), raw kaolinite (Yavuz and Aydin 2006), and spent mushroom waste (Alhujaily et al. 2020). However, the reported adsorption capacity of magnetic nanocomposite of chitosan/SiO_2_/CNTs (Abbasi 2017) towards DB71 is higher than that of ZCPH-2. In a nutshell, Table 4 illustrates the high potential of ZCPH-2 as adsorbent for DB71 and Pb(II) from aquatic environment.

## Conclusions

Three different compositions of zwitterionic copolymer based on [3-(methacryloylamino)propyl]trimethylammonium chloride and sodium 4-vinylbenzenesulfonate as cationic and anionic, respectively, monomers were prepared following a free radical polymerization using bis[2-(methacryloyloxy)ethyl] phosphate as cross-linker. The successful preparation of the different zwitterionic copolymers was confirmed by the FTIR results. The adsorption performance of the different prepared zwitterionic copolymers toward anionic dye and cationic trace metal was evaluated and the promising one was further characterized and used to optimize the adsorption conditions. The zwitterionic copolymer hydrogel with the highest amount of cross-linker (ZCPH-2) was the most promising adsorbent for direct blue 71 (DB71) dye and Pb(II) ions. The presence of several different functional groups and porous structure of ZCPH-2 led to the adsorption process of DB71 and Pb (II) through several interaction pathways. Moreover, the different functional groups onto ZCPH-2 surface constituted small-sized homogenous pitches that are heterogeneous to each other. The uptake of the DB71 dye was observed to be higher in acidic solutions, whereas the highest adsorption of Pb (II) ions occurred at pH 5. The Langmuir adsorption capacities for DB71 and Pb (II) ions were 54.44 and 126.43 mg/g, correspondingly. In summary, the results of this study nominate the application of the zwitterionic copolymer hydrogel ZCPH-2 for removing DB71 and Pb (II) from aquatic environment.

## Data Availability

Data and material will be available if required.
