# Correction to: Preparation and characterization of chemically cross‑linked zwitterionic copolymer hydrogel for direct dye and toxic trace metal removal from aqueous medium

**DOI:** 10.1007/s11356-023-27842-0

**Published:** 2023-05-24

**Authors:** Asmaa A. Koryam, Shaimaa T. El‑Wakeel, Emad K. Radwan, Azza M. Abdel Fattah, Elham S. Darwish

**Affiliations:** 1 Water Pollution Research Department, National Research Centre, 33 El Buhouth St, DokkiGiza, 12622 Egypt; 2 Department of Chemistry, Faculty of Science, University of Cairo, Giza, 12613 Egypt

**Correction to: Environmental Science and Pollution Research**

10.1007/s11356-023-26966-7

The minus sign is missing in Equation 2.

The Original article has been corrected.

